# 

**Published:** 1904-09

**Authors:** 


*The following donations have been received since the publication of the List in June :*

*August 31st, 1904.*

The American Gynecological Society (1) ... ... 1 volume.

Cassell & Company, Limited (2) ... ... ... ... 2 volumes.

L. M. Griffiths (3) ... ... ... ... ... ... ... ... 1 volume.

Rhode Island Medical Society (4) ... ... ... ... 1 volume.

Sir James Sawyer, M.D. (5) ... ... ... ... ... 1 volume.

Editor of *Virginia Medical Semi-Monthly* (6) ... ... 1 volume.

### FIFTY-THIRD LIST OF BOOKS.

The titles of books mentioned in previous lists are not repeated.

The figures in brackets refer to the figures after the names of the donors, and show by whom the volumes were presented. The books to which no such figures are attached have either been bought from the Library Fund or received through the *Journal*.

**Ackland, W. Sanders and T. G.** *Results of the Census in 1901* ... ... ... 1903
**Allbutt, T. C.** ... ... *Notes on the Composition of Scientific Papers* ... ... 1904
*American Gynecological Society; Album of Portraits of Fellows* ... ... (1) 1900
**Appel, E. L. C.** ... *How to Become a Certified Midwife* ... ... ... ... [1904]
**Berry, G. A.** ... ... *Manual of Practical Ophthalmology*... ... ... ... 1904
**Bosanquet, W. C.** ... *Serums, Vaccines, and Toxines in Treatment and Diagnosis* ... ... ... ... ... ... ... (2) 1904
**Bourcart, M.** ... ... *Le Ventre.* Part I. ... ... ... ... ... ... ... ... 1904
**Caiger, F. F.** ... ... *The Diagnosis and Management of Doubtful Cases of Diphtheria* ... ... ... ... ... ... ... ... 1904
**Caldwell, R.** ... ... *The Prevention of Disease in Armies in the Field* ... 1904
**Chapman, W. L.** ... *Auto-Intoxication*... ... ... ... ... ... ... ... (4) 1903
*Chilcott's Descriptive History of Bristol* ... ... ... .. ... (3) 6th Ed. [1844]
**Clarke, W. B.** ... ... *The Meaning of a Modern Hospital* ... ... ... ... 1904
**Dürck, H.** ... ... *Atlas and Epitome of Special Pathologic Histology.* (Ed. by L. Hektoen). ... ... [Vol. II.] 1901
*Encyclopædia Medica* Vol. XIV. ... ... ... ... ... ... ... ... ... ... ... 1904
**Fisher, J. H.** ... ... *Ophthalmological Anatomy* ... ... ... ... ... ... 1904
**French, H.** ... ... *Medical Laboratory Methods and Tests* ... ... ... 1904
**Gadd, H. W.** ... ... *Drugs* ... ... ... ... ... ... ... ... ... ... ... ... 1904
**Glassington, C. W.**... *Golden Rules of Dental Surgery* ... ... ... ... ... [N.D.]
**Gould, G. M.** ... ... *Biographic Clinics.* Vol. II. ... ... ... ... ... ... 1904
**Grimsdale, H. B.** ... *Errors of Refraction* ... ... ... ... ... ... ... ... 1904
**Haines, F. Peterson and W. S.** [Eds.] *Text-Book of Legal Medicine and Toxicology.* Vol. II. ... ... 1904
**Holt, L. E.** ... ... *The Care and Feeding of Children* ... ... 3rd Ed. 1904
**Kelynack, T. N.** ... *The Sanatorium Treatment of Consumption* ... ... 1904
**Lindsay, J. A.**... ... *Diseases of the Lungs and Heart* ... ... ... ... 1904
**Martindale and W. W. Westcott, W.** *The Extra Pharmacopœia.* 11th Ed. (Revised by W. H. Martindale and W. W. Westcott) 1904
**Miles, A. Thomson and A.** *Manual of Surgery.* Vol. II. ... ... ... ... 1904
**Noorden, C. von** ... *Diseases of Metabolism and Nutrition.* Parts IV., V. ... ... ... ... ... ... ... ... ... 1904
**Nuttall, G. H. F.** ... *Blood Immunity and Relationship* ... ... ... ... 1904
**Owen, E.** ... ... *Cleft-Palate and Hare-Lip* ... ... ... ... ... ... 1904
**Paget, S.**... ... ... *What we owe to Experiments on Animals* ... ... ... 1904
,, ... ... ... *The Case against Anti-Vivisection* ... ... ... ... 1904
*Pamphlets.* 29 vols. ... ... ... ... ... ... ... ... ... ... ... ... ... ... [V.D.]
**Peterson and W. S. Haines, F.** [Eds.] *Text-Book of Legal Medicine and Toxicology.* Vol. II. ... ... 1904
**Phillips, C. D. F.** ... *Materia Medica, Pharmacy and Therapeutics: Inorganic Substances* ... ... ... ... 3rd Ed. 1904

**Probyn-Williams, R. J.** *Golden Rules of Anæsthesia* ... ... ... ... ... ... [N.D.]
**Richardson, W. G.** ... *Development and Anatomy of the Prostate Gland* ... 1904
**Riddell, J. S.** ... ... *A Manual of Ambulance* ... ... ... ... ... 5th Ed. 1904
**Sanders and T. G. Ackland, W.** *Results of the Census in 1901* ... ... ... ... 1903
**Sawyer, Sir J.** .. *Practical Medicine* ... ... ... ... ... (5) 4th Ed. 1904
**Searle, R. B.** ... ... *Medical Tuberculosis* ... ... ... ... ... ... ... ... 1904
**Symes, J. O.** ... ... *The Bacteriology of Every-day Practice* ... 2nd Ed. 1904
**Taylor, E. H.** ... *Treatise on Applied Anatomy* ... ... ... ... ... ... 1904
**Thomson and A. Miles, A.** *Manual of Surgery*. Vol. II. ... .. ... 1904
**Thorne, L. T.** ... ... *The "Nauheim" Treatment of Chronic Heart Disease* 1904
**Ward, A. H.** ... ... *Clinical Studies in Syphilis*... ... ... ... ... ... ... 1904
**Watson, J. K.** ... ... *Handbook of Midwifery* ... ... ... ... ... ... ... 1904
**Wenkebach, K. F.** ... *Arhythmia of the Heart*. (Trans. by T. Snowball) 1904
**Westcott, W. Martindale and W. W.** *The Extra Pharmacopœia*. 11th Ed. (Revised by W. H. Martindale and W. W. Westcott) 1904
**Whiteford, C. H.** ... *Anæsthetics in Surgery* ... ... ... ... ... ... 1904
**Whitelocke, R. H. A.** *Football Injuries* ... ... ... ... ... ... ... ... ... 1904
**Wingrave, W.** ... *Adenoids* ... ... ... ... ... ... ... ... ... ... ... 1904
**Yeo, I. B.** ... ... *The Therapeutics of Mineral Springs and Climates* (2) 1904
**Younger, E. G.** ... *Insanity in Every-day Practice* ... ... ... ... ... ... 1904

### TRANSACTIONS, REPORTS, JOURNALS, &c.

American Pediatric Society, Transactions of the ... ... Vol. XV. 1903
Archivio di Ortopedia ... ... ... ... ... ... ... ... ... ... ... ... 1903
Bibliographia Medica ... ... ... ... ... ... ... ... ... Tome III. 1903
British Gynæcological Journal, The ... ... ... ... ... Vol. XIX. 1903-04
Centralblatt für innere Medicin ... ... ... ... ... ... ... ... ... 1902
China Medical Missionary Journal, The ... ... ... ... Vol. XVII. 1903
Clinical Journal, The ... ... ... ... ... ... ... ... ... Vol. XXIII. 1904
Edinburgh Medical Journal, The ... ... ... ... ... ... N.S., Vol. XV. 1904
Hospital, The ... ... ... ... ... ... ... ... ... ... ... Vol. XXXV. 1904
Hospitals—
Middlesex Hospital Reports for 1902 ... ... ... ... ... ... ... 1904
Journal of Medical Research, The ... ... ... ... ... Vol. X. 1903-04
Journal of the Sanitary Institute ... ... ... ... ... ... Vol. XXIV. 1903
Medical Chronicle, The ... ... ... ... ... 4th series, Vol. VI. 1903-04
Medical Library and Historical Journal ... ... ... ... ... Vol. I. 1903
Medical Review of Reviews ... ... ... ... ... ... ... ... ... Vol. IX. 1903
Northumberland and Durham Medical Journal, The ... ... ... ... 1903
Obstetrical Transactions ... ... ... ... ... ... ... ... ... Vol. XLV. 1904
Ohio State Medical Society, Transactions of the ... ... ... ... ... 1903
Philadelphia County Medical Society, Proceedings of the Vol. XXIV. 1903
Progressive Medicine ... ... ... ... ...Vols. II.-IV., 1903; I., II. 1904
Rockefeller Institute of Medical Research, Studies from the ... Vol. I. 1904
Sei-I-Kwai Medical Journal, The ... ... ... ... ... ... ... Vol. XXII. 1903
University of Penna. Medical Bulletin ... ... ... ... ... Vol. XVI. 1904
Virginia Medical Semi-Monthly ... ... ... ... ... ... (6) Vol. VIII. 1904

**Long Fox Memorial Lecture.**—The first of the lectures instituted in memory of the late Dr. Edward Long Fox will be delivered on Friday, November 4th, at 4.30 p.m. at the University College, Bristol.

**University College, Bristol.**—Students of the College have been successful in the following examinations:—University of Cambridge.—*M.B. Examination: Pharmacology and General Pathology*—F. Shingleton Smith. University of London.—*M.B.: Intermediate Examination*—J. W. J. Willcox; *Organic Chemistry only*—V. Moxey, F. S. Scott, L. J. Short. Conjoint Board of the Royal Colleges of Physicians and Surgeons of England.—*First Examination: Chemistry*—S. H. Kingston, E. R. Sircom, B. C. Eskell, H. Templeton. *Practical Pharmacy*—P. S. Connellan, F. C. Morgan, J. F. H. Morgan. *Biology*—E. E. Davies. *Second Examination: Anatomy and Physiology*—E. V. Connellan, R. N. W. Biddulph. *F.R.C.S.: Final Examination*—F. R. Walters. Society of Apothecaries.—*Surgery*—J. E. Jones. *Medicine and Forensic Medicine*—A. H. Hughes. *Forensic Medicine*—J. E. Jones. Faculty of Physicians and Surgeons, Glasgow. Dental Board. *First Examination*—C. H. Taylor.

Arrangements are being made for the establishment of a **post-graduate course of instruction**, and we have no doubt that the scheme will meet with general approval and will be of much value to candidates for the higher examinations. It is proposed that a two or three months' course of lectures, with special clinical opportunities both at the Royal Infirmary and General Hospital, shall be at once commenced for any qualified candidates who may need such instruction. The details of this course will shortly be announced.

**Royal Hospital for Sick Children and Women.**—Dr. T. A. Green has been elected as Out-patient Surgeon.

**The late Dr. George Charles Wigan, of Portishead.**—A beautiful stained-glass window of three lights, in memory of the late Dr. G. C. Wigan and Mrs. Wigan, has been placed in Portishead Parish Church, and was dedicated by the rector on July 16th.

